# Supplementary material for: Tunable anisotropic van der Waals films of 2M-WS2 for plasmon canalization
Source: Nat Commun. 2024 Mar 23;15:2623. doi: 10.1038/s41467-024-46963-9 (PMC10960863; doi:10.1038/s41467-024-46963-9)
Supplement: Supplementary file 1 — Supplementary Information [file 41467_2024_46963_MOESM1_ESM.pdf]

Supplementary Information for

Tunable anisotropic van der Waals films of 2M-WS_2 for plasmon canalization

Qiaoxia Xing[1], Jiasheng Zhang[1], Yuqiang Fang[2,3], Chaoyu Song[1], Tuoyu Zhao[4,5], Yanlin Mou[1], Chong Wang[6,7], Junwei Ma[1], Yuangang Xie[1], Shengyang Huang[1], Lei Mu[1], Yuchen Lei[1], Wu Shi[4,5], Fuqiang Huang[2,3,8]* and Hugen Yan[1]*

[1] State Key Laboratory of Surface Physics, Key Laboratory of Micro and Nano-Photonic Structures (Ministry of Education), Shanghai Key Laboratory of Metasurfaces for Light Manipulation, and Department of Physics, Fudan University, Shanghai 200433, China.

[2] State Key Laboratory of High Performance Ceramics and Superfine Microstructure, Shanghai Institute of Ceramics, Chinese Academy of Sciences, Shanghai 200050, China.

[3] School of Materials Science and Engineering, Shanghai Jiao Tong University, Shanghai 200240, China.

[4] State Key Laboratory of Surface Physics and Institute for Nanoelectronic Devices and Quantum Computing, Fudan University, Shanghai 200433, China.

[5] Zhangjiang Fudan International Innovation Center, Fudan University, Shanghai 201210, China.

[6] Centre for Quantum Physics, Key Laboratory of Advanced Optoelectronic Quantum Architecture and Measurement (MOE), School of Physics, Beijing Institute of Technology, Beijing 100081, China.

[7] Beijing Key Lab of Nanophotonics & Ultrafine Optoelectronic Systems, School of Physics, Beijing Institute of Technology, Beijing 100081, China.

[8] Beijing National Laboratory for Molecular Sciences and State Key Laboratory of Rare Earth Materials Chemistry and Applications, College of Chemistry and Molecular Engineering, Peking University, Beijing 100871, China.

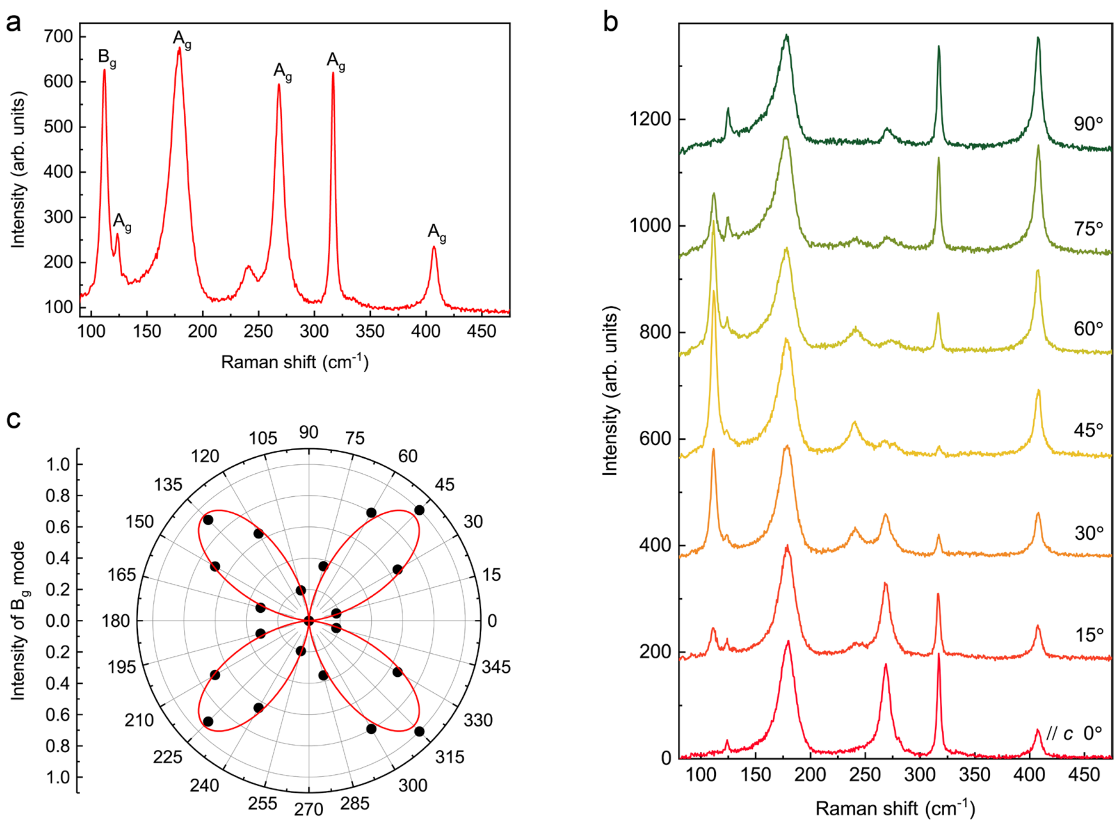

Supplementary Fig. 1 | Raman spectra of a bulk 2M-WS_2 crystal measured using a Horiba HR LabRam system with a laser wavelength of 532 nm. a Raman spectrum of a bulk 2M-WS_2 crystal, where seven Raman active phonons belonging to A_g and B_g modes are observed[1-3]. **b** Polarized Raman spectra of the bulk 2M-WS_2 crystal. In the measurement process, a parallel polarization configuration for the polarizer and analyzer was adopted, and we rotated the sample from 0° to 180°, with the incident light polarized along the *c*-axis defined as 0°. **c** Angle-dependent intensity of the B_g mode, this result is consistent with the corresponding Raman selection rule $I(\mathrm{B_g}) \propto \sin^2 2\theta$, as indicated in the reference[1].

Supplementary Note 1: Infrared spectra fitting procedures

To fit the spectra of unpatterned film of 2M-WS_2, the complex optical conductivity is given by:

$$\sigma(\omega) = \frac{i}{\pi}\frac{D}{(\omega + i\Gamma)} + \frac{i}{\pi}\sum_{N}\frac{\omega S_{\mathrm{inter},N}}{(\omega^2 - \omega_{\mathrm{inter},N}^2 + i\omega\Gamma_{\mathrm{inter},N})} \quad (N = 1, 2, 3, 4, 5, 6) \quad \text{(S1)}$$

where D is the Drude weight, $S_{\mathrm{inter},N}$ is the Nth interband spectrum weight,

$\omega_{\text{inter},N}$ is the frequency of the Nth interband resonance. Γ and $\Gamma_{\text{inter},N}$ are scattering rates of Drude response and the Nth interband transition, respectively. The first term is the Drude response, six Lorentz functions which represent interband transitions in the second term are also used. Together with equation (1) in the main paper, we fit the spectra in Supplementary Fig. 2a-b to extract the real and imaginary parts of the conductivity, as shown in Supplementary Fig. 2c.

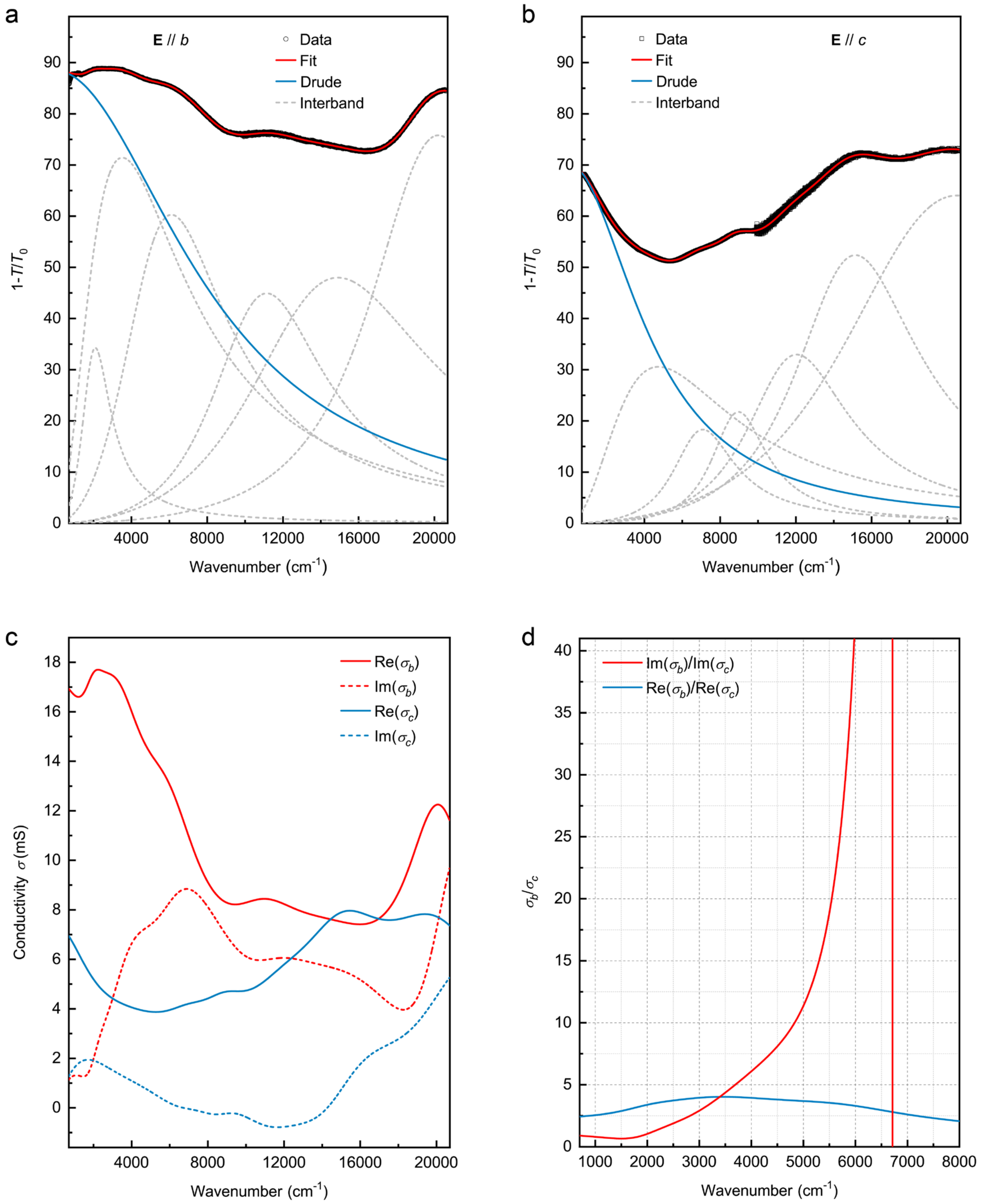

Supplementary Fig. 2 | The extinction spectra and the fitted conductivity of the film in Fig. 1 in the main paper. a-b The extinction spectra as well as the corresponding fitting curves along the two principal axes of the 2M-WS_2 film in Fig. 1 in the main paper. **c** Extracted real and imaginary parts of the conductivity of the 2M-WS_2 film along the *b*- and *c*-axis at room temperature. **d** The frequency dependence of the optical conductivity ratio between two axes extracted from **c**, with relatively large σ_b''/σ_c'' above 3000 cm^{-1}.

To extract the parameters from the plasmon spectra in Fig. 2a-b in the main paper, the following model for the optical conductivity $\sigma(\omega)$, which also takes into account interband transitions, is employed:

$$\sigma(\omega) = i\frac{f}{\pi}\frac{\omega W}{\left(\omega^2 - \omega_{\mathrm{p}}^2 + i\omega\Gamma_{\mathrm{p}}\right)} + i\frac{f}{\pi}\sum_N \frac{\omega S_{\mathrm{inter},N}}{\left(\omega^2 - \omega_{\mathrm{inter},N}^2 + i\omega\Gamma_{\mathrm{inter},N}\right)} \quad \text{(S2)}$$

where ω_{p} and Γ_{p} ($\omega_{\mathrm{inter},N}$ and $\Gamma_{\mathrm{inter},N}$, N=1, 2) are the frequency and linewidth of the plasmon (interband transition), W and $S_{\mathrm{inter},N}$ are the spectral weights of plasmon and interband transitions, respectively, and f is the filling factor of the disk array. Figure 2d in the main paper and supplementary Fig. 3 show representative fitting curves for the spectra, with the extracted plasmon frequencies and linewidths shown in Figs. 3a and 3b in the main paper, and plasmon spectra weights shown in Supplementary Fig. 5b.

To extract the plasmon frequencies of the skew ribbons, similar to the case of the disks for which the measurement range is about 800-12000 cm^{-1}, two effective interbands were taken into account. For some of the spectra of the skew ribbons, only the range of 800-8000 cm^{-1} was measured owing to the relatively low plasmon frequency. Given the measurement range as well as the plasmon dominance in the spectra and some of the interband becoming indistinguishable after patterning, in addition to one Lorentz peak for plasmon, 1-2 effective interband peaks were sufficient in the fitting to extract the plasmon frequency.

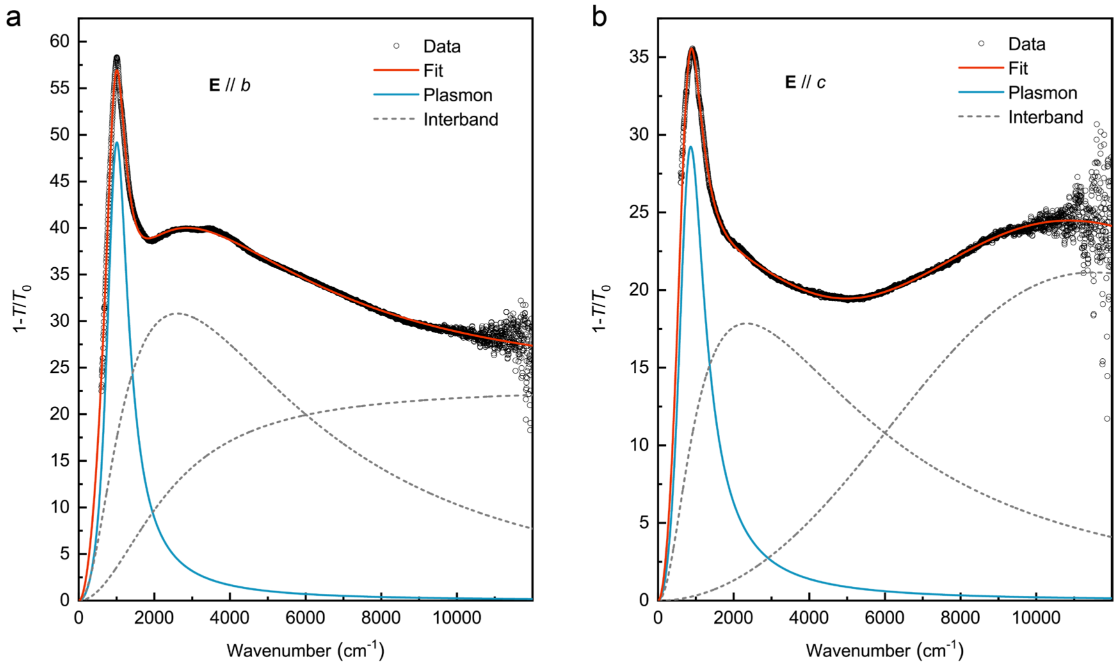

Supplementary Fig. 3 | An example showing the extinction spectra fitting results of the 2.5 μm disk in Fig. 2a in the main paper.

Supplementary Note 2: Finite element simulation

The far-field spectra in Fig. 2c in the main paper were simulated using a frequency-domain solver for Maxwell equations based on the finite element software (Comsol Multiphysics). A disk of 2M-WS_2 in the center of an air domain was surrounded by a perfectly matched layer. The plane wave with the electric field polarized along *x*- or *y*-directions was set as the background field. The extinction cross-section was calculated, and the convergence of the simulation was monitored while refining the meshes. To facilitate comparison with experimental results, extinction spectra were obtained according to the relation between extinction cross-section and transmittance[4], as shown in Fig. 2c of the main paper. To avoid interference and save the computer memory, the diamond substrate with a thickness of about 1 mm used in the experiment was not taken into account in the simulation.

The electric field distribution $|\mathbf{E}|$ of the surface plasmons excited by a *z*-oriented electric dipole located 10 nm above a 2M-WS_2 film was simulated. The 2M-WS_2 film was represented by a conductive surface with the anisotropic optical conductivity extracted from the unpatterned thin film in Fig. 1 in the main paper, and the field

monitor was placed 5 nm below the 2M-WS_2 surface. As shown in Supplementary Fig. 4, the elliptical electric field distribution $|\mathbf{E}|$ gradually elongates with increasing plasmon frequency. This behavior arises as a consequence of the increasing ratio of σ_b''/σ_c'' and the reduction in losses attributed to the decreased real part of the conductivities along the two axes with increase of the frequency. The change in conductivity versus frequency is displayed in Supplementary Fig. 2d.

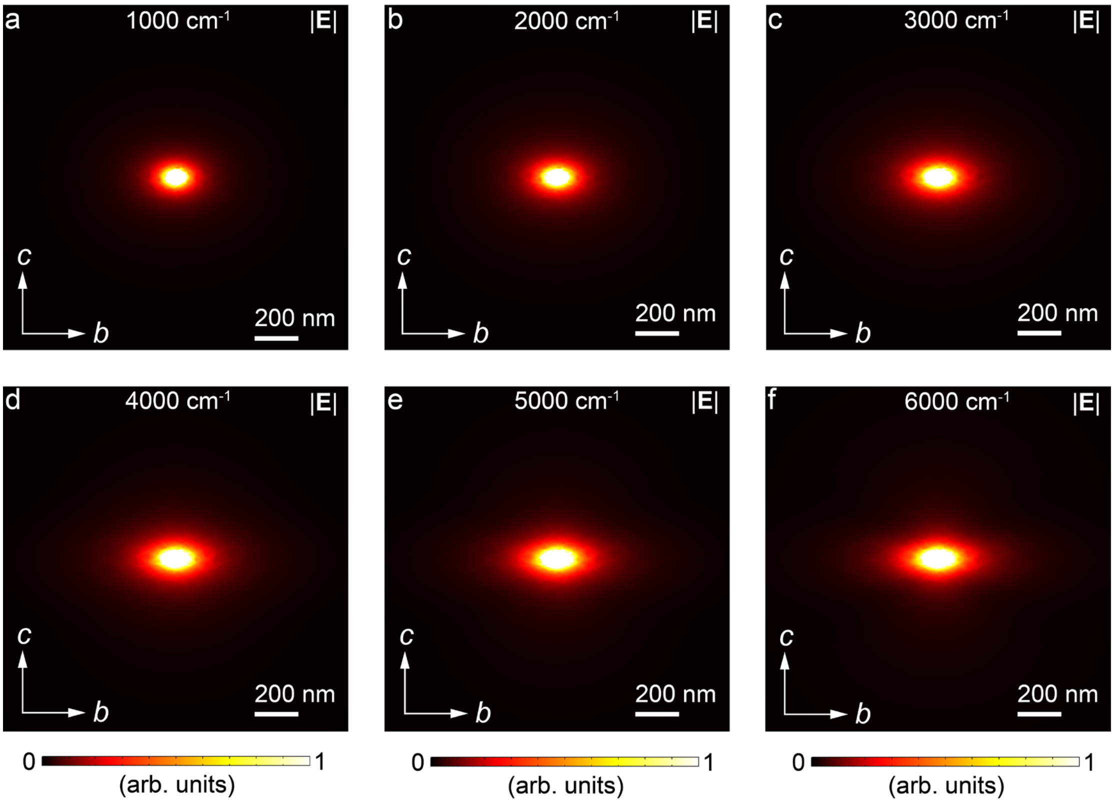

Supplementary Fig. 4 | Near-field intensity distribution |E| of the surface plasmon at various frequencies excited by a *z*- oriented electric dipole.

Supplementary Note 3: Calculation of the loss function -Im(1/*ε*)

The loss function can be derived through the standard random-phase approximation (RPA). Here we don't neglect the retardation effect. The RPA dielectric function can be expressed as[5]:

$$\varepsilon_{RPA}(\mathbf{q},\omega) = 1 - D_l(\mathbf{q},\omega)\chi^{(0)}_{j_l j_l}(\mathbf{q},\omega) \tag{S3}$$

$D_l(\mathbf{q},\omega)$ is the part of the photon propagator:

$$D_l(\mathbf{q},\omega) = e^2 q'/(2\varepsilon_0\omega^2)\,,q' = \sqrt{q^2 - \varepsilon(\omega/c)^2} \tag{S4}$$

ε is the relative dielectric constant of the surrounding environment. $\chi^{(0)}_{j_l j_l}(\mathbf{q},\omega)$ represents the average longitudinal current-current correlation function within the free system. Because the relation between current-current correlation function $\chi_{j_l j_l}(\mathbf{q},\omega)$ and the density-density correlation function $\chi_{\rho\rho}(\mathbf{q},\omega)$ is as follows:

$$q^2\chi_{j_l j_l}(\mathbf{q},\omega) = \omega^2\chi_{\rho\rho}(\mathbf{q},\omega) \tag{S5}$$

$$\chi_{j_l j_l}(\mathbf{q},\omega) = \frac{\chi^{(0)}_{j_l j_l}(\mathbf{q},\omega)}{1 - D_l(\mathbf{q},\omega)\chi^{(0)}_{j_l j_l}(\mathbf{q},\omega)} \tag{S6}$$

$$\chi_{\rho\rho}(\mathbf{q},\omega) = \frac{\chi^{(0)}_{\rho\rho}(\mathbf{q},\omega)}{1 - v(\mathbf{q})\chi^{(0)}_{\rho\rho}(\mathbf{q},\omega)} \tag{S7}$$

where $v(\mathbf{q}) = e^2/(2\varepsilon_0 q)$, and $\chi^{(0)}_{\rho\rho}(\mathbf{q},\omega)$ is the 2D polarizability. Thus we get the following relation:

$$q^2\chi^{(0)}_{j_l j_l}(\mathbf{q},\omega) = \omega^2\chi^{(0)}_{\rho\rho}(\mathbf{q},\omega) \tag{S8}$$

According to the Supplementary information of reference[6], the relation between the 2D polarizability and the longitudinal conduction σ_L can be expressed as:

$$\chi^{(0)}_{\rho\rho}(\mathbf{q},\omega) = \frac{\sigma_L}{ie^2}\frac{q^2}{\omega} \tag{S9}$$

Therefore, considering a sample sandwiched between two dielectrics with the relative dielectric constants ε_i $(i = 1, 2)$, the RPA dielectric function including the retardation effect is as follows:

$$\varepsilon_{RPA}(\mathbf{q},\omega) = 1 + \frac{i\sigma_L}{\varepsilon_0\omega}\frac{q_1' q_2'}{\varepsilon_2 q_1' + \varepsilon_1 q_2'} \tag{S10}$$

with $q_i' = \sqrt{q^2 - (\omega/c)^2\varepsilon_i}$, $(i = 1, 2)$[7]. In the nonretarded limit ($c \longrightarrow \infty$), Eq. S10 reduces to the known 2D dielectric function in reference[8].

Supplementary Note 4: Linewidth and spectrum weight

Supplementary Figure 5a presents the fitted linewidth for spectra in Fig. 2 in the main paper, which increases linearly from ~170 cm^{-1} to ~3400 cm^{-1} with the increase of the

wave vector, except for plasmons oscillating along the *c*-axis at room temperature. The linear fit (light gray dashed dot line) shows a good agreement with the data, and this behavior is a result of the combined effects of the retardation (lower frequency range) and interband transitions (higher frequency range). The linewidth of plasmons in disks scales as $1/d$ (d is the diameter of the disk) when the retardation is taken into account[9]. The plasmons along the *c*-axis at room temperature exhibit the largest linewidth, due to the larger Drude scattering rate and interband linewidth at room temperature compared to liquid nitrogen temperature. Supplementary Figure 5b presents the fitted spectra weight versus the wave vector **q**, the spectra weight first increases and then decreases. Electric dipole radiation at low frequencies and interband Landau damping at high frequencies contribute to the reduction in spectrum weight[10, 11].

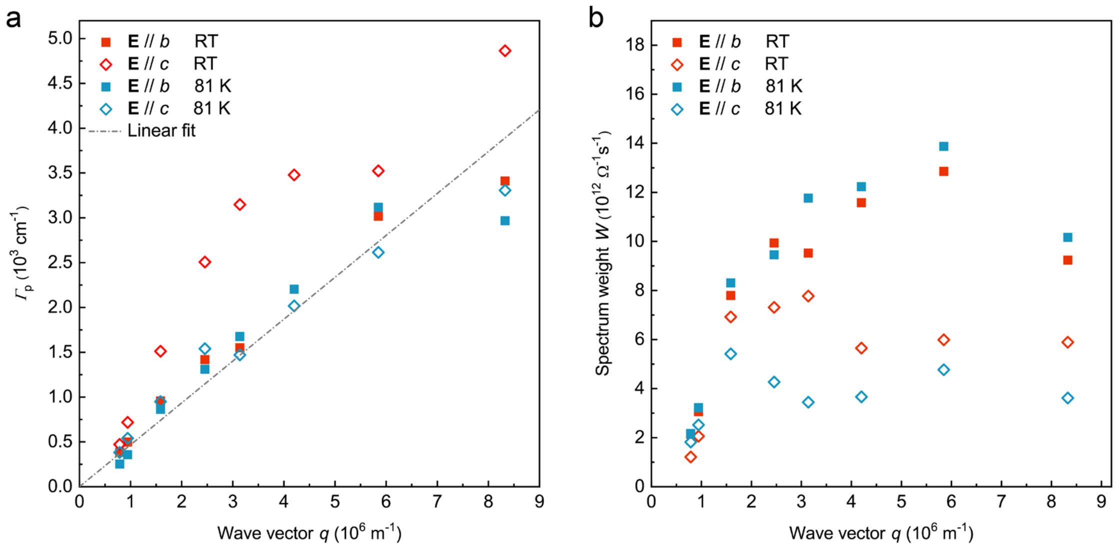

Supplementary Fig. 5 | Linewidths and spectra weights of plasmons extracted from Fig. 2a-b in the main paper. a Extracted linewidth of the plasmons in Fig. 2a-b in the main paper. **b** Extracted spectrum weight of the plasmons in Fig. 2a-b in the main paper.

Supplementary Note 5: Isofrequency contours obtained through the loss function

To validate the isofrequency contours obtained from the experiment in Fig. 4 in the

main paper, we plot isofrequency contours based on plasmon dispersions calculated from the loss function using the conductivities along different directions of the film, as shown in Supplementary Fig. 6. To extract the maximum of the loss function for a direction with the skew angle of θ relative to the b-axis, the longitudinal conductivity $\sigma_L = \sigma_b \cos^2\theta + \sigma_c \sin^2\theta$ (σ_b and σ_c are the 2D conductivities along the b- and c-axis, respectively) was used in Eq. S10. Considering the contribution of interband transitions to the loss functions at relatively high frequency range where the plasmons were damped, and the actual feasible frequency ranges of plasmons along different directions in experiment, the wave vector $\mathbf{q}$ was cutoff to $1.4 \times 10^7\ m^{-1}$ due to the nearly indiscernible plasmon strength along all directions up to that wave vector (see Supplementary Fig. 7). It is important to note that the direction dependence of the plasmon dispersions in Fig. 4a in the main paper is not as pronounced as that in Supplementary Fig. 6a. Since the interaction between the ribbons in the retardation regime typically results in a redshift of the plasmon, which is not taken into account in the loss function calculated dispersion in Supplementary Fig. 6a. For 2M-WS_2 with the conductivity along the b-axis larger than that of the c-axis, the interaction-induced plasmon frequency redshift along the b-axis is greater than that along the c-axis.

By accounting for the field interaction between the structures, the effective polarization and polarizability are expressed as[12]:

$$\mathbf{P} = \alpha^* \mathbf{E} \tag{S11}$$

$$\alpha^* = \frac{\alpha}{1 - \alpha S} \tag{S12}$$

where α represents the polarizability of a single structure without considering the interaction, and S denotes the retarded dipole sum depending on the structure separation, arrangement and operating frequency[13]. The radiative dipole coupling between the ribbons modifies the polarizability, and thus the plasmon frequency. Since the long-range dipolar interaction leads to the plasmon redshift[14, 15], the equivalent optical conductivity of the unpatterned film related to the plasmon frequency can be treated as a fraction of the original when the radiative dipole

coupling between the structures is taken into account. In Fig. 4a in the main paper, the dispersions displayed as solid lines were obtained through the maxima of the loss functions, with the conductivity along the *b*-axis being scaled by a factor of 0.43 to fit the experimental data and to guide the eye. Therefore, the anisotropy of the isofrequency contours in Fig. 4b in the main paper is slightly compromised due to the coupling of the structures. Specifically, given the aforementioned dipole-dipole coupling induced plasmon dispersion redshift after patterning when the conductivity along *b*-axis is included in the total conductivity, a larger momentum q is needed for the same plasmon frequency in experiment. Thus, the isofrequency contours seem to be elongated, especially along the *b*-axis in Fig. 4b in the main paper, compared to the loss function calculated one shown in Supplementary Fig. 6b (considering only the intrinsic conductivity of 2M-WS_2 films).

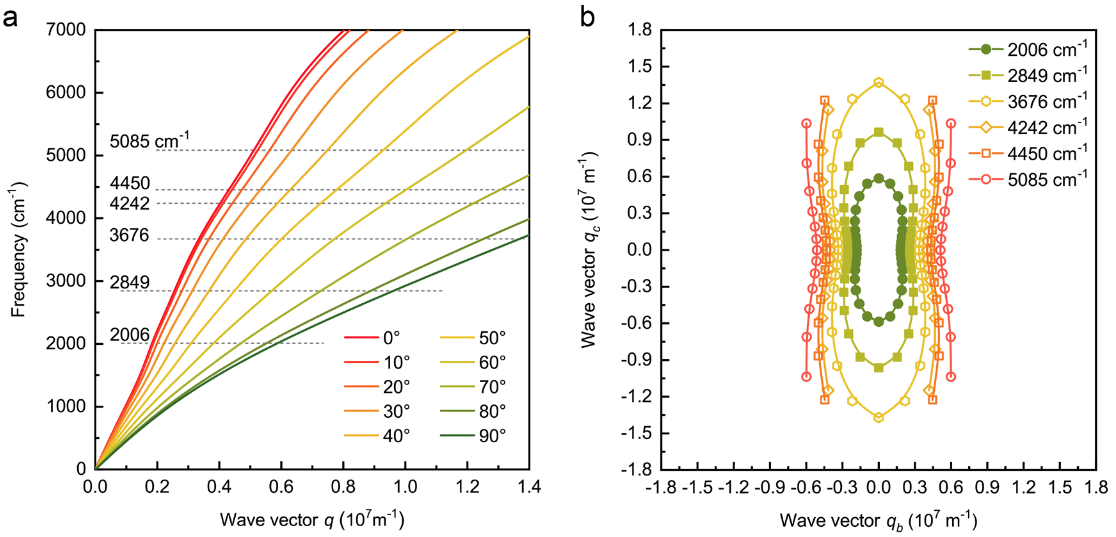

Supplementary Fig. 6 | Plasmon dispersions and isofrequency contours based on the loss function by using the optical conductivity extracted from the 2M-WS_2 film in Fig. 1 in the main paper. a Dispersions of the plasmon in 2M-WS_2 with various skew angles obtained from the maxima of the loss functions. **b** Isofrequency contours of the 2M-WS_2 plasmons constructed according to the frequency cutting lines in **a**.

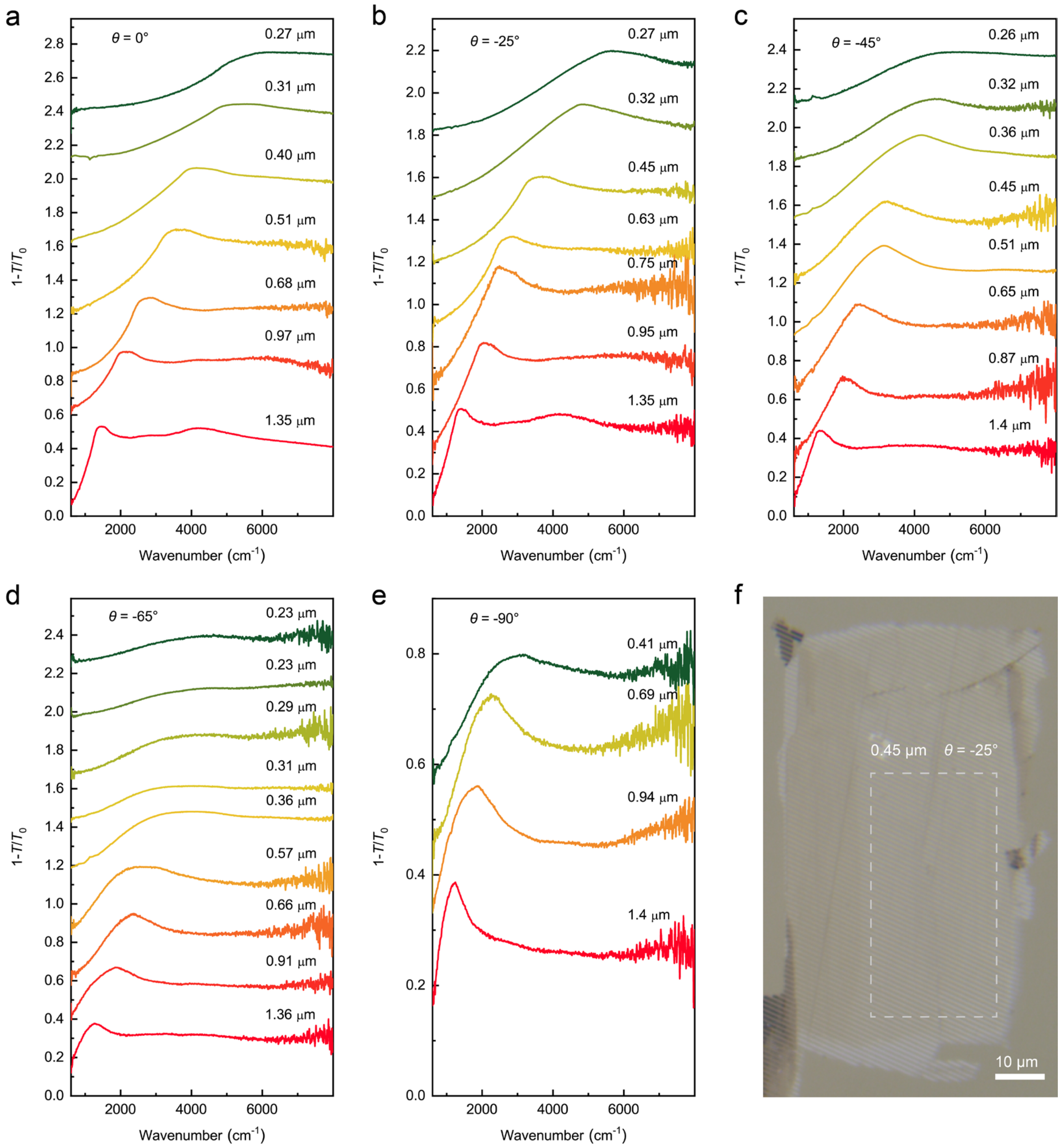

Supplementary Fig. 7 | Extinction spectra and a typical optical image of 2M-WS_2 skew ribbons. a-e Extinction spectra of 2M-WS_2 skew ribbons with a set of skew angles and ribbon widths, where the electric field of the polarized infrared light is perpendicular to the ribbons. The spacing is ~1.3 times the ribbon width. **f** Optical image of a typical skew ribbon array with a skew angle of -25° and a ribbon width of 0.45 μm.

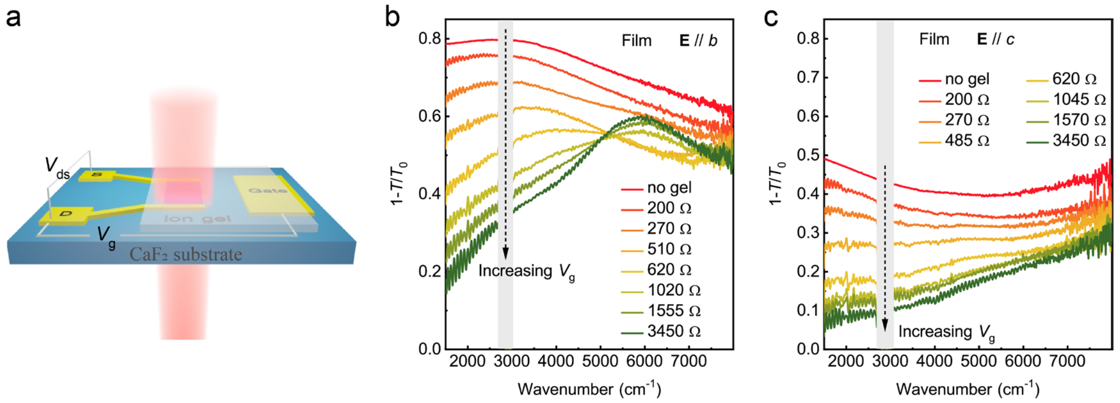

Supplementary Fig. 8 | Extinction spectra of tunable 2M-WS_2 film through ion-intercalation. a An illustration of infrared spectrum measurement of an ion intercalated sample. **b** and **c** Extinction spectra of a 2M-WS_2 film along the *b*- and *c*-axis with Li^+ intercalation-controlled resistances, respectively. The thickness of the film is about 35 nm. Due to the strong absorption of the ion-gel below 1500 cm^{-1} and other region with shade of gray in the figures, these frequency regimes are not displayed.

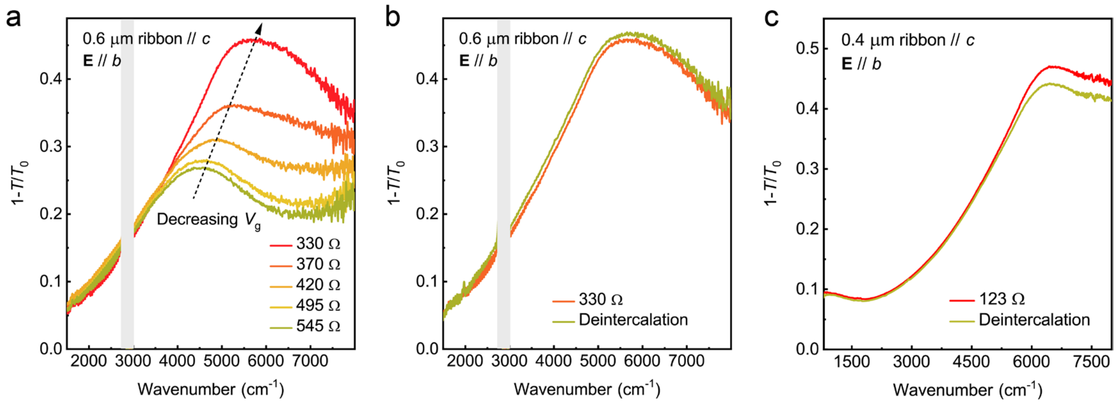

Supplementary Fig. 9 | Reversibility of the ion intercalation. a Plasmon (*b*-axis) evolution in a ribbon array with ribbon width of 0.6 µm under gate-controlled Li^+ deintercalation. **b** Comparison of spectra between the sample with newly dropped ion-gel and after four times of Li^+ intercalation and deintercalation process. **c** Comparison of spectra between the sample before the intercalation and after the deintercalation process under gate-controlled deionized water.

Supplementary Note 6: Calculated intercalation dependent isofrequency contours at the frequency of 3000 cm^{-1}

With increasing intercalated ion concentration with a water droplet, the evolution of spectra of a 2M-WS_2 film along the *c*- and *b*-axis is displayed in Supplementary Fig. 10a and Supplementary Fig. 11a, respectively. As shown in Supplementary Fig. 10b-k and Supplementary Fig. 11b-k, through joint fitting to keep the interband parameters synchronous (except for the linewidth of the interband for the highest frequency along the *b*-axis), the intercalation dependent Drude weights were extracted. As presented in Fig. 5g in the main paper, Drude weight along the *c*-axis (*b*-axis) decreases proportionally and eventually decreases to 0.1 (0.15) times of the original with increasing intercalation duration. Supplementary Fig. 12 traces the ratio of the Drude weight at each intercalation step over the Drude weight before intercalation along the *c*-axis versus the corresponding value along the *b*-axis. It firstly undergoes a one-to-one linear decrease and then the decrease along the *b*-axis slows down after the Drude weights along the two principal axes decrease to 0.4 time of the original values. Therefore, we plotted intercalation dependent isofrequency contours at the frequency of 3000 cm^{-1} by tracing the maximum of the loss function, which based on the intrinsic conductivities extracted from the film in Fig. 1d but with only Drude weights decreasing from 1 to 0.5 times of the original values.

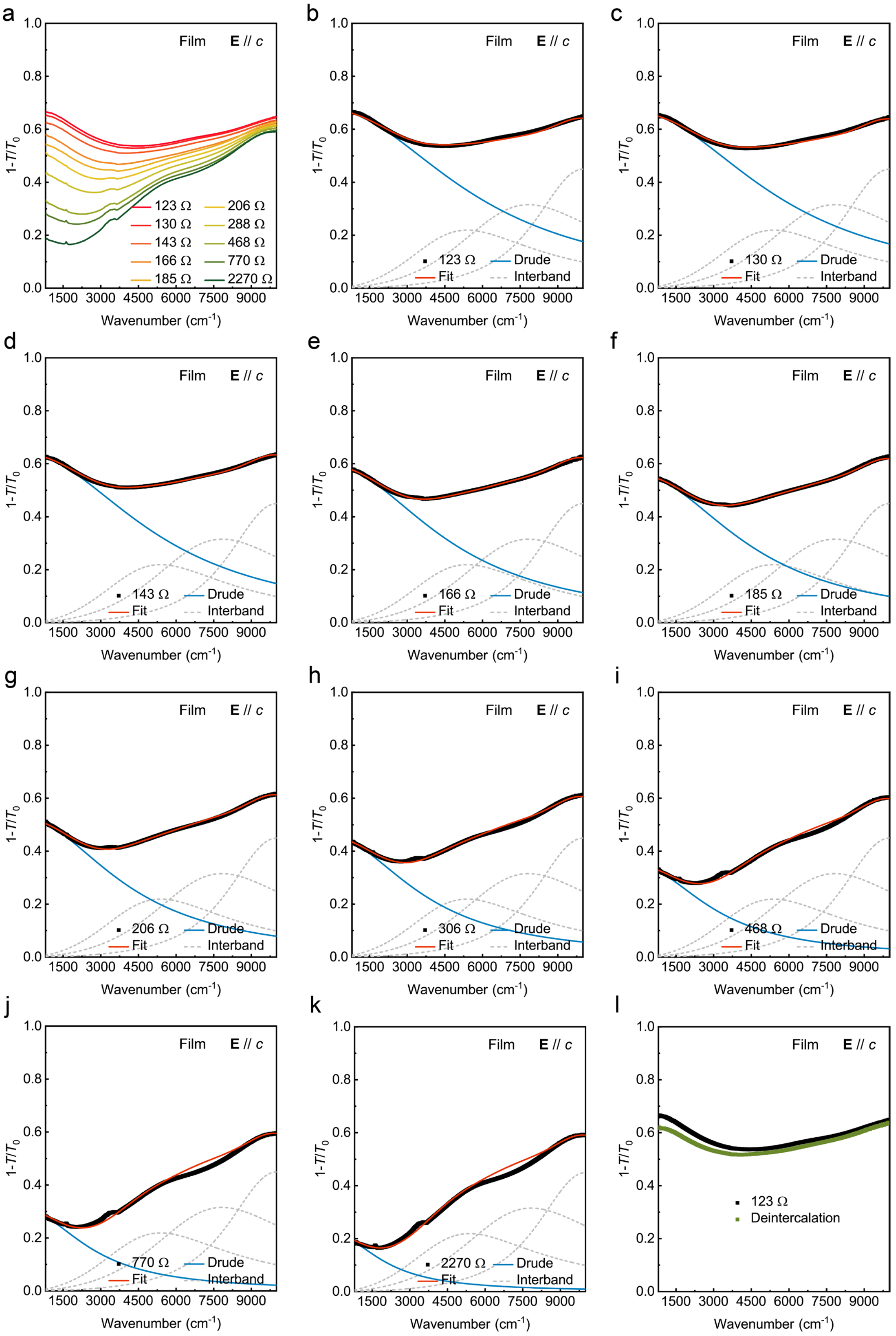

Supplementary Fig. 10 | a Extinction spectra of an intercalated 2M-WS_2 film through deionized water with light polarized along the *c*-axis. The thickness of the film is about 40 nm. **b-k** Fitting results of the spectra in **a**. **l** Comparison of spectra

between the sample before the intercalation and after the deintercalation process.

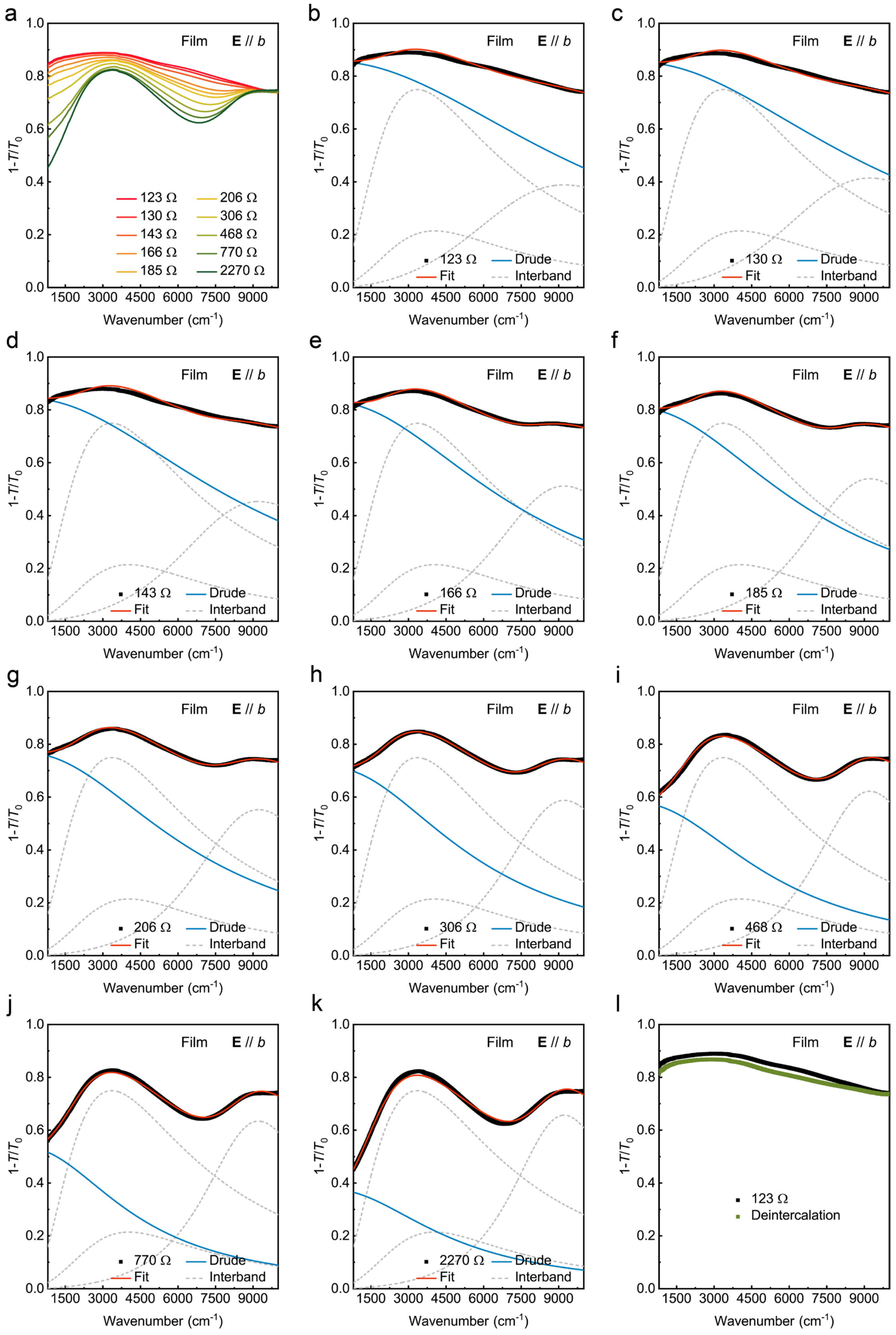

Supplementary Fig. 11 | a *b*-axis polarized extinction spectra of the same

intercalated 2M-WS_2 film in Supplementary Fig. 10. **b**-**k** Fitting results of the spectra in **a**. **l** Comparison of spectra between the sample before the intercalation and after the deintercalation process.

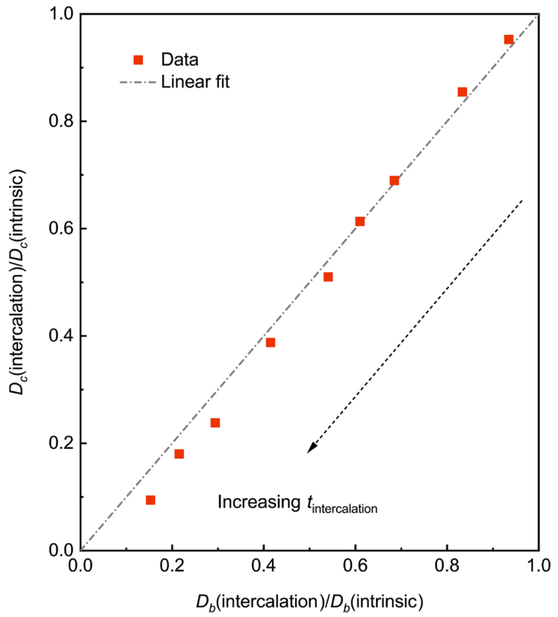

Supplementary Fig. 12 | The ratio of the Drude weight at each intercalation step over the Drude weight before intercalation along the *c*-axis versus the corresponding value along the *b*-axis. The light gray dashed dot line is a linear fitting with slope of 1.

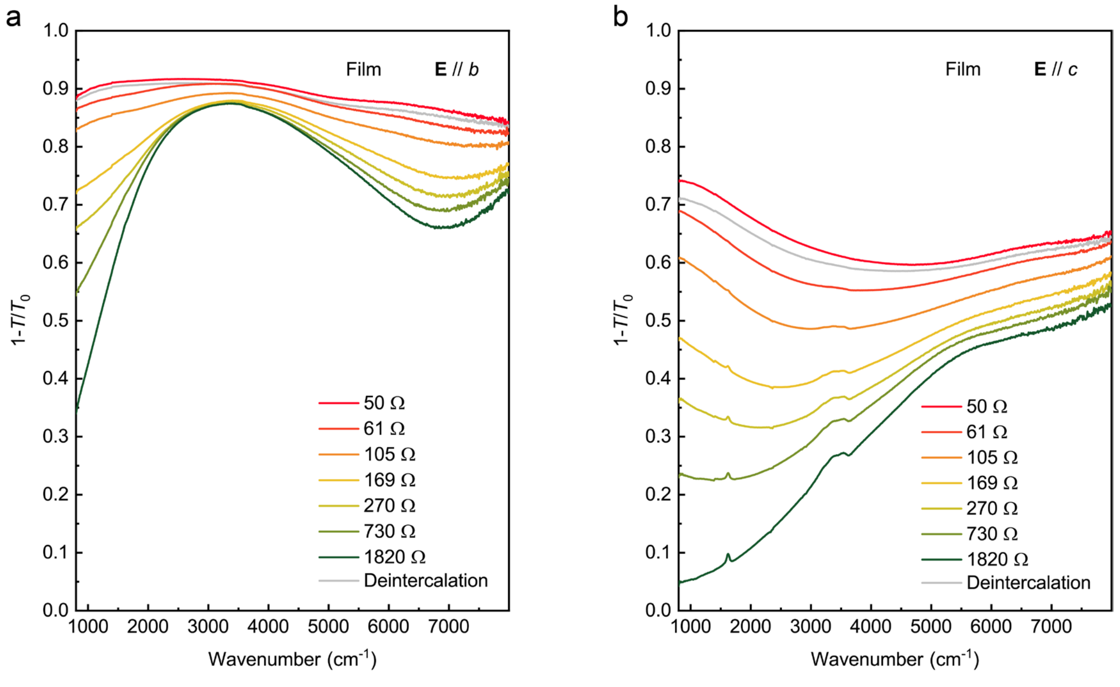

Supplementary Fig. 13 | Reproducibility of the ion intercalation through gate-controlled deionized water. Extinction spectra of another 2M-WS_2 film with light polarization along the *b*- and *c*-axis with ion intercalation and deintercalation. The thickness of the film is about 45 nm.

Supplementary References

1. L. Li, W. Wang, P. Gong, X. Zhu, B. Deng, X. Shi, G. Gao, H. Li and T. Zhai, 2D GeP: An Unexploited Low‐Symmetry Semiconductor with Strong In‐Plane Anisotropy. Advanced Materials **30**, 1706771 (2018).
2. F. Baniasadi. Structure Characterization and Electronic Properties Investigation of Two-Dimensional Materials. Virginia Polytechnic Institute and State University, (2021).
3. Z. Lai *et al.*, Metastable 1T'-phase group VIB transition metal dichalcogenide crystals. Nat. Mater. **20**, 1113 (2021).
4. V. Amendola, R. Pilot, M. Frasconi, O. M. Marago and M. A. Iati, Surface plasmon resonance in gold nanoparticles: a review. J. Phys.: Condens. Matter **29**, 203002 (2017).
5. G. Gómez-Santos, Thermal van der Waals interaction between graphene layers. Phys. Rev. B **80**, 245424 (2009).
6. C. Wang, S. Huang, Q. Xing, Y. Xie, C. Song, F. Wang and H. Yan, Van der Waals thin films of WTe_2 for natural hyperbolic plasmonic surfaces. Nat. Commun. **11**, 1158 (2020).
7. M. S. Ukhtary and R. Saito, Effective impedance of two-dimensional metal with retardation effect. J. Phys.: Condens. Matter **33**, 185302 (2021).
8. T. Stauber, Plasmonics in Dirac systems: from graphene to topological insulators. J. Phys.: Condens. Matter **26**, 123201 (2014).
9. I. Zoric, M. Zach, B. Kasemo and C. Langhammer, Gold, Platinum, and

Aluminum Nanodisk Plasmons: Material Independence, Subradiance, and Damping Mechanisms. ACS Nano **5**, 2535 (2011).

10. C. Ropers, D. J. Park, G. Stibenz, G. Steinmeyer, J. Kim, D. S. Kim and C. Lienau, Femtosecond light transmission and subradiant damping in plasmonic crystals. Phys. Rev. Lett. **94**, 113901 (2005).
11. M. B. Ross and G. C. Schatz, Radiative effects in plasmonic aluminum and silver nanospheres and nanorods. J. phys., D, Appl. phys. **48**, 184004 (2015).
12. V. G. Kravets, A. V. Kabashin, W. L. Barnes and A. N. Grigorenko, Plasmonic Surface Lattice Resonances: A Review of Properties and Applications. Chem. Rev. **118**, 5912 (2018).
13. S. Zou, N. Janel and G. C. Schatz, Silver nanoparticle array structures that produce remarkably narrow plasmon lineshapes. J. Chem. Phys. **120**, 10871 (2004).
14. R. Adato, A. A. Yanik, J. J. Amsden, D. L. Kaplan, F. G. Omenetto, M. K. Hong, S. Erramilli and H. Altug, Ultra-sensitive vibrational spectroscopy of protein monolayers with plasmonic nanoantenna arrays. Proc. Natl. Acad. Sci. U.S.A. **106**, 19227 (2009).
15. A. O. Pinchuk and G. C. Schatz, Nanoparticle optical properties: Far- and near-field electrodynamic coupling in a chain of silver spherical nanoparticles. Materials Science and Engineering: B **149**, 251 (2008).
